# A randomised clinical trial comparing outcomes of a single digit volar plate injury — Buddy loops versus dorsal thermoplastic orthosis in a neutral position: study protocol

**DOI:** 10.1186/s12891-023-06192-5

**Published:** 2023-02-27

**Authors:** Sarah Walsh, Paul Fahey, Karen Liu

**Affiliations:** 1 Sydney Hospital Hand Unit, 8 Macquarie St, Sydney, NSW 2000 Australia; 2 Western Sydney University – School of Health Sciences, Translational Health Research Institute, Sydney, Australia; 3 Department of Rehabilitation Sciences The Hong Kong Polytechnic University, Hong Kong SAR, China

**Keywords:** Hand therapy, Volar plate injury, Randomised clinical trial, Hand injury, Buddy loops, Thermoplastic orthosis

## Abstract

**Background:**

Volar plate injuries are a common hand injury and complications associated with this injury such as a fixed flexion deformity, persistent pain and oedema can have a significant impact on a person’s function. The literature reports these injuries are treated using various splinting materials such as thermoplastic, in varying degrees of proximal interphalangeal joint flexion or buddy loops. Despite volar plate injuries being reported as common, optimal non-surgical treatment of these injuries remains unclear.

This study aims to investigate whether a dorsal blocking orthosis in a neutral position (0^0^) is more effective than buddy loops for a volar plate injury to the proximal interphalangeal joint in preventing a fixed flexion deformity, reducing pain, managing oedema, and promoting function.

**Methods:**

This study is a single-centre, prospective parallel-group, single blinded (assessor), randomised clinical trial. Patients between 18–65 years, who have sustained a volar plate injury to a single digit, have adequate cognitive functioning and give written informed consent will be invited to participate in this study. Patients will be randomised to either the control group where they will be fitted with buddy loops and commence early active motion exercises or the experimental group where they will receive a dorsal thermoplastic orthosis in a neutral position and commence early active motion exercises. The primary outcome measure is passive proximal interphalangeal joint extension and secondary outcome measures include passive range of motion, total passive motion, active range of motion, total active motion, grip strength, oedema, pain, function and adherence to treatment. Assessments will be completed until 8 weeks following commencement of treatment. The sample size calculation indicates that 23 patients is required in each group. With an expected dropout rate of 25% a total of 32 patients will be enrolled in each group.

**Discussion:**

This study will assist in trying to improve treatment of volar plate injuries and assist in reducing complications associated with volar plate injuries, potentially reducing the need for prolonged hand therapy.

**Trial registration:**

This trial has been registered with the Australian New Zealand Clinical Trials Registry (ACTRN12622001425785p). Ethical approval has been granted by the South Eastern Sydney Local Health District ethical committee (2022/ETH01697).

**Supplementary Information:**

The online version contains supplementary material available at 10.1186/s12891-023-06192-5.

## Background

Volar plate injuries are a common injury [1] and are often sustained during sport, whereby the finger is pushed back, stretching the volar plate [2]. This hyper-extension force can often cause an avulsion of the volar aspect of the middle phalanx where the volar plate inserts or can cause a dorsal dislocation of the Proximal Interphalangeal Joint (PIPJ) [3].

Volar plate injuries are reported to be a common injury in the hand [4]. The Eaton classification is used in the literature to describe the severity of volar plate injuries to the PIPJ. Eaton type I injuries are described as a hyperextension injury whereby there is either a partial or complete avulsion of the volar plate from the middle phalanx and may or may not include a bone fragment; Eaton type II are described as a dorsal dislocation with a complete avulsion of the volar plate and a bilateral split in the collateral ligament; Eaton type III are described as a fracture dislocation where the volar plate is avulsed from its insertion and creates a disruption at the base of the middle phalanx on the volar surface. These injury types are subdivided into stable (IIIa) or unstable (IIIb) [5].

Although volar plate injuries are common, the optimal way to conservatively treat them is still not clear [5]. Historically, volar plate injuries have been treated by splinting the joint in varying degrees of PIPJ flexion (10^o^ – 30°) [6, 7] using Plaster of Paris, an aluminium or plastic orthosis [7–10]. Protected mobilisation has also been demonstrated using a dorsal thermoplastic orthosis that blocks the PIPJ from completely extending but allows the patient to flex the injured digit by undoing the Velcro straps [11]. Although early protected motion has been shown to be safe and assists in preventing stiffness into flexion, the risk of a Fixed Flexion Deformity (FFD) remains due to the soft tissues around the joint healing in a shortened position due to the positioning of the joint in a flexed position [12].

An alternative in the literature, that also allows protected mobilisation, is buddy loops [6]. Buddy loops involve ‘buddying’ the injured digit to a border digit using a strap or tape. It provides support and protection while allowing early motion. Research suggests that buddy loops can be effective in producing good Range of Motion (ROM) outcomes for volar plate injuries [13], but the evidence reported is largely retrospective or they have a large proportion of children and adolescents included in the cohort.

Another alternative treatment is to immobilise the joint in a neutral position using a dorsal thermoplastic orthosis. A recent study by Stanley, Seifman [7] compared treatment of volar plate injuries by splinting the PIPJ in 30° of flexion and compared it to splinting the joint in neutral (0°). The study concluded that splinting the joint in a neutral position led to better extension of the PIPJ and did not lead to issues with instability or hyper extensibility. However, the research is retrospective, and the groups were uneven with 105 participants in the group with the PIPJ in flexion and only 20 were the PIPJ was positioned in neutral, reducing the statistical power of the study.

Positioning the PIPJ in a neutral position has also been demonstrated in cadaveric studies. A study completed by Tyser, Tsai [14] found that volar plate injuries with up to a 20% bony defect remained stable in a neutral position during testing, whereas a bony defect of up to 40% was reported to be variable and the threshold for PIPJ stability. This has been reinforced by Caravaggi, Shamian [15] who found that collateral ligament and volar plate injuries alone as well as fractures of less than 30% of the joint surface were unlikely to be unstable.

Looking at the reported treatment interventions, the research suggests that while splinting the PIPJ in a flexed position does provide joint stability, it may also be a significant contributor to the most reported complication of a FFD [16].

Buddy loops are noted as a cost effective and efficient method for treatment of volar plate injuries [6]. They promote early active motion however, it is unclear if they provide adequate positioning to prevent a FFD, particularly in the presence of oedema, pain or in Eaton IIIa classified injuries.

Based on the research available, splinting the PIPJ in a neutral position may result in superior outcomes as it positions the joint in an optimal resting position and allows protected early active motion. Using buddy loops to treat volar plate injuries has been reported to provide good Active Range of Motion (AROM) outcomes due to the early and easy commencement of early active motion, however, could still lead to the development of a FFD due to the natural resting posture of the digit. Due to the limitations in the current literature, further research is required to determine the optimal way to treat these injuries.

The aim of this study is to investigate whether a dorsal blocking orthosis (splint) in a neutral position (0°) is more effective than buddy loops in the treatment of a volar plate injury. Specific hypotheses will address; splinting the PIPJ in a neutral position produces a) a lower average extension contracture of a FFD than splinting in buddy loops; b) lower mean pain scores than buddy loops; c) lower mean oedema measure than buddy loops; and d) splinting the PIPJ in a neutral position results in changed adherence to treatment compared to buddy loops.

## Methods

The study is a randomised controlled, single-centre, parallel, single blinded (assessor) trial.

### Participants

All patients who attend the Sydney Hospital Hand Clinic between March 2023 - September 2024 and meet the selection criteria will be invited to participate in the study.

Patients will be invited to participate in this study if they are aged 18–65 years, are diagnosed with a closed volar plate injury to a single digit (Eaton classification I, II, IIIa), do not require surgical intervention, have adequate cognitive functioning, in that they are able to understand written and verbal information regarding their injury and participation in the study, have their first treatment at Sydney Hospital within 14 days of sustaining their injury and can give written informed consent.

Patients will be excluded from the study if they have a concomitant tendon or nerve injury, other fracture, vascular injury, or open injury, a previous or existing condition that affects the same or contralateral digit resulting reduced AROM and/or pain.

### Sample size

A mean difference of 10° in passive PIPJ extension scores between the control and experimental groups is deemed to be clinically relevant. Based on a quality improvement project on volar plate injuries at Sydney Hospital, a standard deviation of 10° in passive PIPJ extension scores is estimated [17]. To be able to detect this difference with 90% power at the 5% significance level, 23 participants will be required in each group. Factoring in a dropout rate of 25% brings the total to 32 participants in each group.

At Sydney Hospital Hand Clinic, there were 149 presentations for volar plate injuries in the period between 22/06/2017 – 18/12/2018. It is anticipated that there will be a 50% uptake of patients consenting to be involved in the study. Therefore, recruitment and data collection is anticipated to take 18 months.

### Randomisation

Enrolment and randomisation will be conducted after obtaining signed informed consent from a patient.

To ensure sufficient and balanced treatment groups, there will be 64 random draws of ‘orthosis’ or ‘buddy loops’ in blocks of four. That is, there will be two thermoplastic orthoses and two buddy loops in each block of four. Each of the 64 random draws will be printed on a card. Each card will be placed in a separate sealed opaque envelope. The envelopes will be numbered on the outside in draw order. Therapists will be instructed on the importance of drawing the envelopes in correct sequence and adhering to the treatment written on the card. The chief investigator or one of the assigned treating hand therapists will take the next envelope in the sequence.

**Fig. 1:**
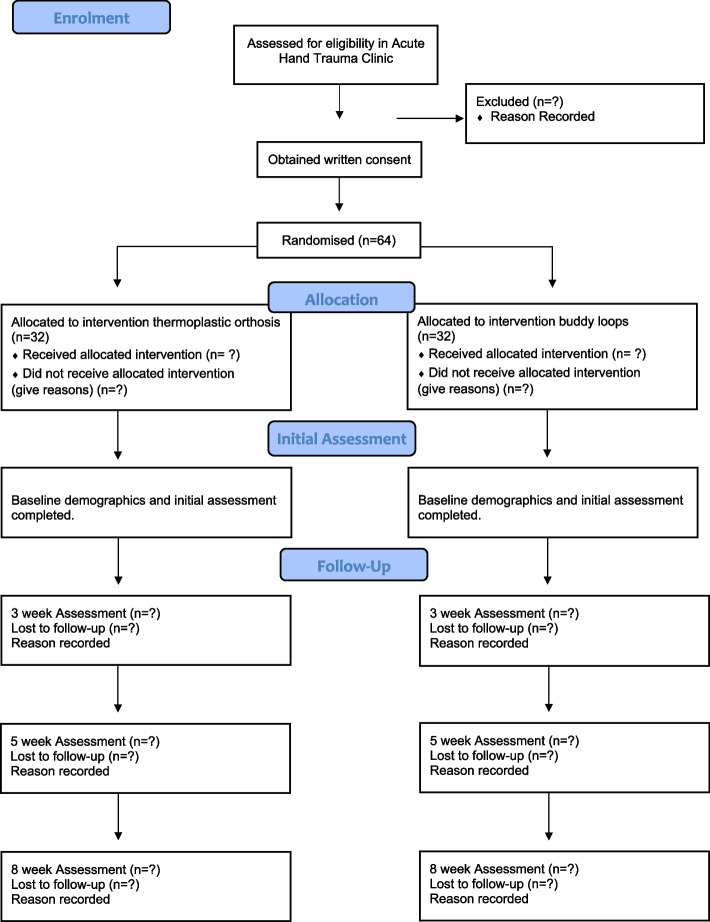
CONSORT Flow Chart

If participants consent, they will be randomised to either the experimental or the control group. The participant flow is shown in Fig. 1. Each eligible patient presenting to the hand clinic will be invited to participate in the study.

### Intervention

The intervention as part of this research will continue for eight weeks. In the experimental group, patients will be treated with a dorsal blocking orthosis in a neutral (0° extension) position. In the control group, patients will be treated using buddy loops, where the injured digit is ‘buddied’ to a border digit for support.

**Fig. 2:**
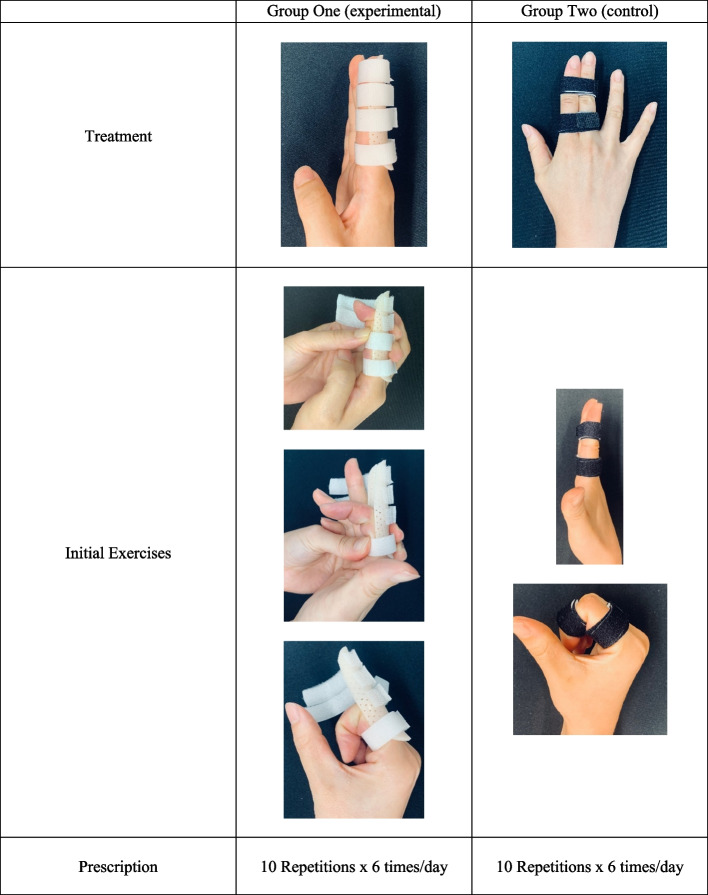
Intervention of experimental and control groups

Participants in both groups will commence protected early AROM exercises (Fig. 2). For the experimental group, this will entail commencing active isolated Distal Interphalangeal Joint (DIPJ) and PIPJ flexion and extension exercises and then composite flexion and extension exercises within the orthosis. In the control group, this will entail actively flexing and extending all the joints of the injured digit within the buddy loop. Patients will be asked to complete 10 repetitions, 6 times per day. Exercises will be progressed as per treatment guidelines (Appendix 1).

At three weeks after the commencement of treatment, if clinically indicated, both groups will start to wean out of their orthosis or buddy loops but continue to wear them for sleeping and ‘at risk’ activities. At five weeks’ post commencement of treatment, patient will discharge their orthosis or buddy loops if clinically appropriate.

Treatment will be progressed as clinically indicated as per the treatment guideline (Appendix 1) that has been developed to improve consistency among treating therapists. The treatment guideline will be provided to treating therapists and provide clinical scenarios that they might encounter such as ‘good passive but reduced active PIPJ flexion’ and recommended exercises they can implement to address this impairment.

### Data collection

Baseline demographic data will be collected, and the initial assessment will be completed by a blinded hand therapist. Data collection will then be completed at weeks 3, 5 and 8, which will coincide with planned treatment progression (Table 1).

**Table 1:** Data collection table

	Initial	3 Weeks	5 Weeks	8 Weeks
Demographics	✓			
**Primary outcome measure**				
PIPJ PROM extension		✓	✓	✓
**Secondary outcome measures**				
MCPJ AROM (^0^) Injured	✓	✓	✓	✓
MCPJ AROM (^0^) Uninjured	✓			
PIPJ AROM (^0^) Injured	✓	✓	✓	✓
PIPJ AROM (^0^) Uninjured	✓			
DIPJ AROM (^0^) Injured	✓	✓	✓	✓
DIPJ AROM (^0^) Uninjured	✓			
TAM (^0^) Injured	✓	✓	✓	✓
TAM (^0^) Uninjured	✓			
MCPJ PROM (^0^) Injured		✓	✓	✓
MCPJ PROM (^0^) Uninjured		✓		
PIPJ PROM (^0^) Uninjured		✓		
DIPJ PROM (^0^) Injured		✓	✓	✓
DIPJ PROM (^0^) Uninjured		✓		
TPM (^0^) Injured		✓	✓	✓
TPM (^0^) Uninjured		✓		
Pain (NRS)	✓	✓	✓	✓
PRWHE	✓	✓	✓	✓
Oedema (cm)	✓	✓	✓	✓
Grip Strength (Kg) Injured			✓	✓
Grip Strength (Kg) Uninjured			✓	
Adherence		✓		
RTW/Usual Everyday Activities	✓	✓	✓	✓
Complications		✓	✓	✓

### Outcome measure

Outcome measures will be gathered at baseline and weeks 3, 5 and 8 after the commencement of treatment (Table 1).

The primary outcome measure will be passive PIPJ extension. Passive PIPJ extension will be assessed in a standardised way as described by American Society of Hand Therapists (ASHT) [18]. Assessment of Passive Range of Motion (PROM) with a goniometer has been shown to be consistently reliable when standardised methods are implemented [18].

Secondary outcome measures for this study will include AROM and PROM of other joints, grip strength, oedema, pain level, function and treatment adherence will be captured.

AROM of all the joints of the injured digit including the Metacarpal Phalangeal Joint (MCPJ), PIPJ and DIPJ. This will be compared to the contralateral uninjured digit and will be undertaken using a single goniometer. In addition to AROM, Total Action Motion will be calculated and compared to the uninjured contralateral digit as per the ASHT guidelines [18].

PROM of all the joints of the injured digit will be compared to the contralateral uninjured digit and will be undertaken using a single goniometer. PROM can provide information about a joint’s capacity for motion [19] and ensure the home exercise program is tailored specifically to the patients’ impairments. In addition, Total Passive Motion will be calculated and compared to the uninjured contralateral digit [18].

Grip strength will be assessed using a standardised Jamar Dynamometer and adopting a standardised testing procedure as per the ASHT guidelines [19]. A single score of maximum effort will be recorded for the injured and uninjured side [20].

Oedema will be assessed using standardised testing procedure as described by ASHT guidelines. A standardised tape measure will be used to take a circumferential measurement of the PIPJ [21] and compared to the contralateral uninjured side.

Pain will be assessed using a Numerical Rating Scale. The patient will be asked to rate their pain between ‘0' and ‘10', with ‘0' describing ‘no pain’ and ‘10’ used to describe ‘worst pain imaginable’. Pain will also be assessed using the pain section of the Patient Rated Wrist and Hand Evaluation (PRWHE), which has been demonstrated to be a valid and reliable assessment of patient rated function and disability and complements the Numerical Rating Scale to assess pain in a holistic way [22].

Function will be assessed using the PRWHE, which has been shown to have good levels of evidence for validity, reliability [23] and responsiveness to trauma [24]. Return to work/everyday activities status will be determined by asking the patient if they have returned to their pre-injury roles, modified role or not at all. The adverse impact of hand injuries on return to work and everyday activities have been reported in the literature [25].

Adherence to treatment will be assessed via a questionnaire developed based on the work of SandfordBarlow [26]. The questionnaire asks the same key questions regarding adherence to treatment such as, if the splint has been removed, how often it may have been removed and why it may have removed but adapted slightly to reflect the different diagnostic group. This will be measured at the three-week assessment point, after this stage, patients will cease wearing the orthosis/buddy loop full time and commence a weaning process.

### Blinding

Trained assessors will be blinded from group allocation and different to the treating therapist. Prior to the assessment, the patient will be asked to remove their orthosis or buddy loop and will be discouraged from discussing any form of their treatment to the assessing therapist to ensure the blinding.

### Data analysis

Analysis will commence with descriptive statistics. Histograms and descriptive statistics will be used to review the distribution of continuous variables for outliers, skewness and other potential problems. Counts and percentages will be used to review categorical variable for small categories. Scatterplots and side-by-side boxplots will be employed to visually check for associations between variables. Outliers will be checked for data errors and, if legitimate, adjusted for using Winsorizing or trimming. Data transformations will be used to address skewness if required.

Independent samples t-tests will be used review the statistical significance of these differences in means at follow-up. Analysis of covariance (ANCOVA) will be used to adjust for any differences between groups at baseline. Repeated measures analysis of variance (ANOVA-RM) or linear mixed models will be used to model differences between treatment groups over time. Data will be analysed using an ‘intention to treat’ analysis.

### Adverse events

Complications associated with volar plate injuries to the PIPJ (Eaton type I, II, IIIA) are commonly reported in the literature and include FFD of the PIPJ, PIPJ extension lag, persistent pain, persistent oedema, reduced flexion and reduced grip strength.

These common complications are usually managed through hand therapy intervention. The hand therapists at Sydney Hospital Hand Unit are experienced therapists able to identify and treat these complications. In addition, all treating and assessing hand therapists will be provided with training that will include identification and treatment of these impairments. A treatment guideline has been developed to assist with managing these impairments.

Less common complications associated with volar plate injuries to the PIPJ include instability and re-dislocation of the PIPJ. These complications are not anticipated to occur as joints that are unstable are more likely to be associated with Eaton Type IIIb and are excluded from this study.

If an unanticipated event occurs these will be reported back to the researcher by face- to-face communication; email or phone. The researcher is on site, which should make reporting of incidents from a research point of view easy and efficient. The assessing therapists will also be able to identify complications at each assessment point using the assessment sheet; if a re-dislocation occurs an incident report should be lodged by the treating/assessing therapist. If an incident report is made the researcher will be notified via email. This is as per standard clinical practice within the hand unit at Sydney Hospital.

In terms of clinical management of less common complications these will be managed as per standard practice. There are clear management guidelines established within the hand unit at Sydney Hospital and include; reviewing the patient with a senior hand therapist in the team; organising a medical review for the patient by liaising with a person in the medical team in the hand clinic, by contacting the on-call hand surgeon and organising an urgent review and by lodging an incident report via the online reporting system.

A document will be used to record common and uncommon complications in both groups and reviewed by the investigative team every 3 months. Any strong imbalance between treatment groups will be reported to the clinical head of the Hand Unit and the Ethics Committee who may terminate the trial.

## Conclusions

This study will add valuable knowledge to the field of hand therapy regarding the treatment of volar plate injuries. If this study can determine an optimal way to treat volar plate injuries it could assist in reducing common complications associated with these injuries, allow people to return to their usual everyday activities earlier and reduce the amount of hand therapy intervention required to treat volar plate injuries.

## Supplementary Information

**Additional file1:** **Appendix 1.** Treatment guideline for treating Hand Therapists.

## Data Availability

Data will be stored according as required by the ethics committee and will be available from the authors on request. Please contact Sarah Walsh for data information related to the study.

## Abbreviations

AROM: Active range of motion
ASHT: American society of hand therapists
DIPJ: Distal interphalangeal joint
FFD: Fixed flexion deformity
MCPJ: Metacarpal phalangeal joint
PIPJ: Proximal interphalangeal joint
PROM: Passive range of motion
PRWHE: Patient rated wrist and hand evaluation
