# Supplementary material for: A randomised clinical trial comparing outcomes of a single digit volar plate injury — Buddy loops versus dorsal thermoplastic orthosis in a neutral position: study protocol
Source: BMC Musculoskelet Disord. 2023 Feb 27;24:149. doi: 10.1186/s12891-023-06192-5 (PMC9969704; doi:10.1186/s12891-023-06192-5)
Supplement: Supplementary file 1 — Additional file1: Appendix 1. Treatment guideline for treating Hand Therapists. [file 12891_2023_6192_MOESM1_ESM.docx]

| Appendix 1. Treatment guideline for treating Hand Therapists | | | | |
| --- | --- | --- | --- | --- |
|  | | | | |
| **Part A:**  **Clinical Scenario One** | Reduced active and passive flexion | | | |
| Composite passive flexion exercises | 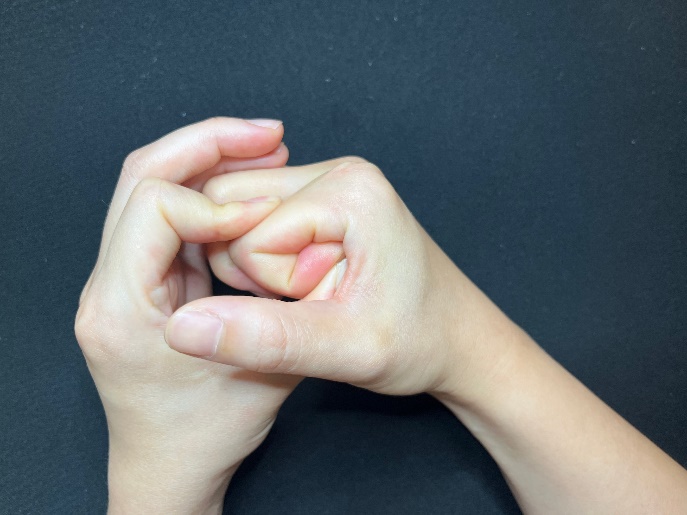 | | Bend all 3 finger joints until a gentle stretch is felt | 3 repetitions x 30^+^seconds x 3/day |
| Flexion Strap | 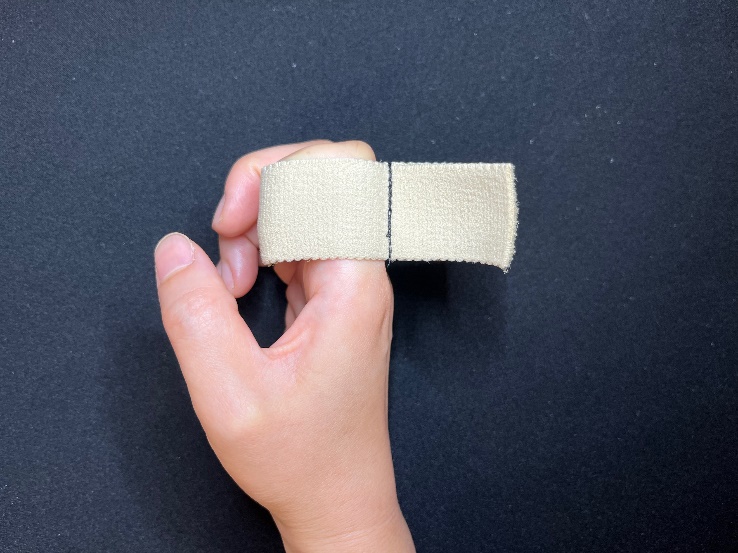 | | Secure a flexion strap that provides a gentle stretch to the patients PIPJ and DIPJ | x3/day as able |
| **Part B:**  **Clinical Scenario Two** | Good passive but reduced active flexion | | | |
| Squeezing a sponge | 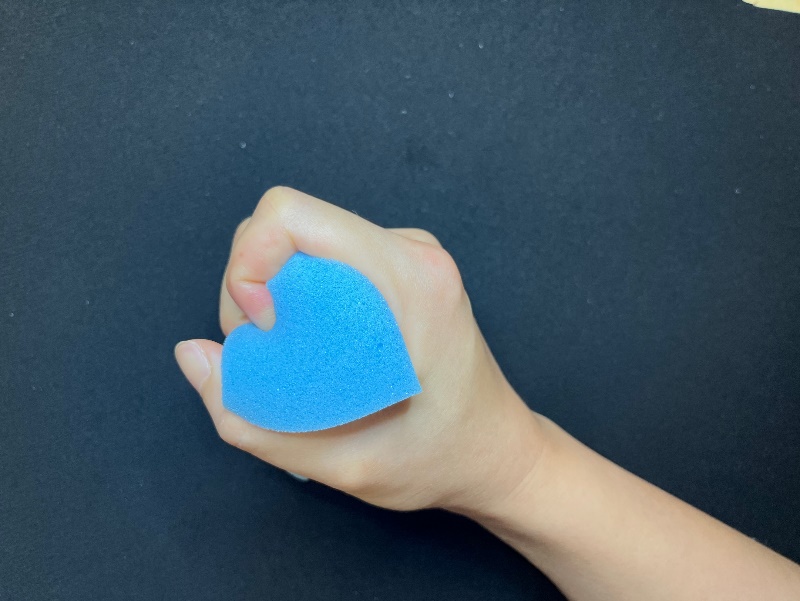 | | Squeeze the sponge by trying to bend all finger joints as far as you can | 10-20 max repetitions (stop earlier if fatigued), hold for 2-3 seconds x2 sets x 2/day |
| Squeezing theraputty | 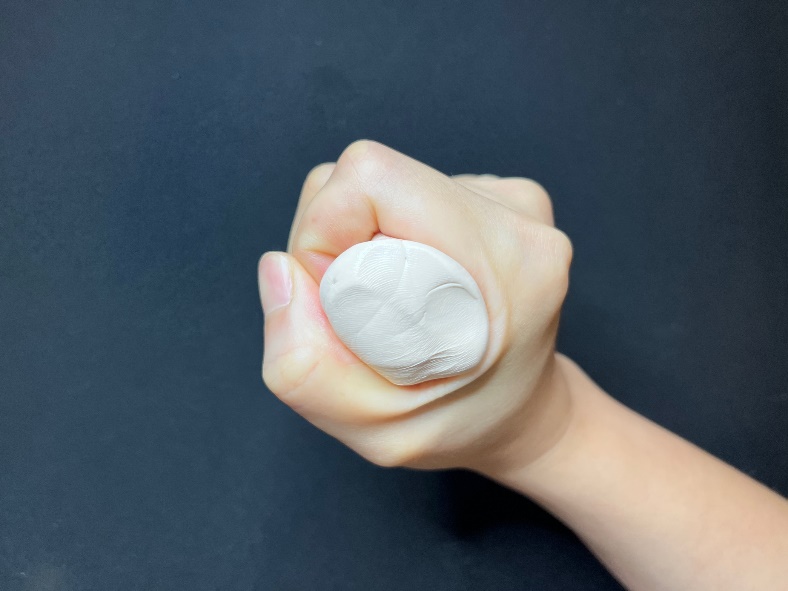 | | Squeeze the Thera putty as far as possible by bending all 3 finger joints as far as you can | 5-10 mins max  (stop earlier if fatigued) x2/day |
| **Part C: Clinical Scenario Three** | Reduced active and passive extension | | | |
| Passive PIPJ extension exercises | 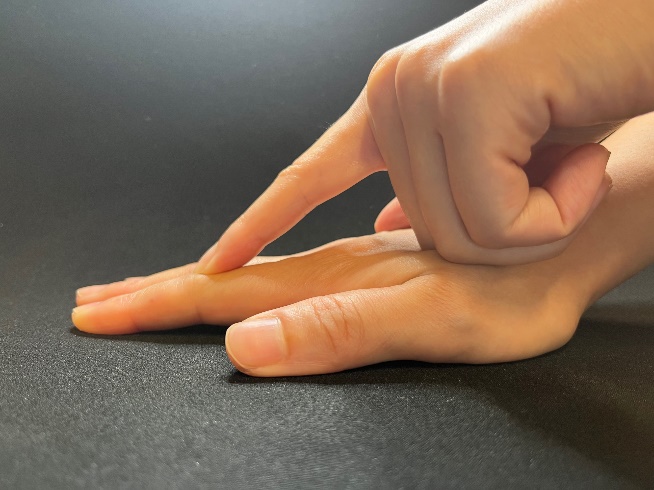 | Place the hand on a flat surface and gently press down over the PIPJ until a gentle stretch is felt  NB a pen can be places under the DIPJ to increase leverage/stretch of the PIPJ | | 3 repetitions x 30^+^ seconds x3/day |
| Neoprene stall | 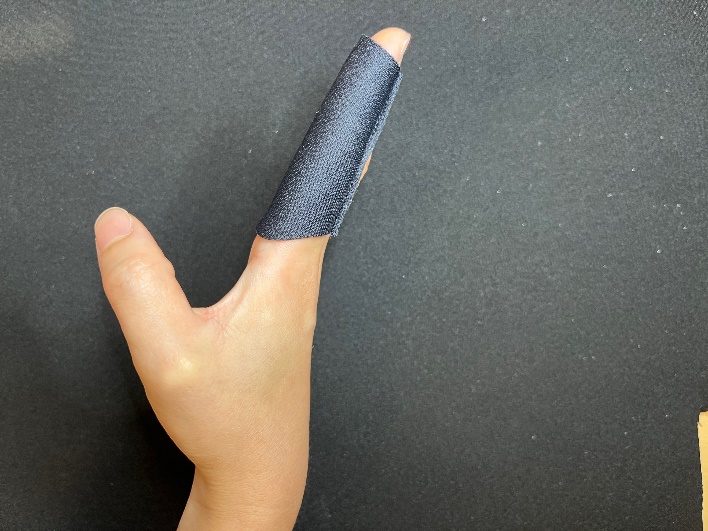 | Fabricate a neoprene stall with gentle pressure | | Day and/or night |
| Extension orthosis | 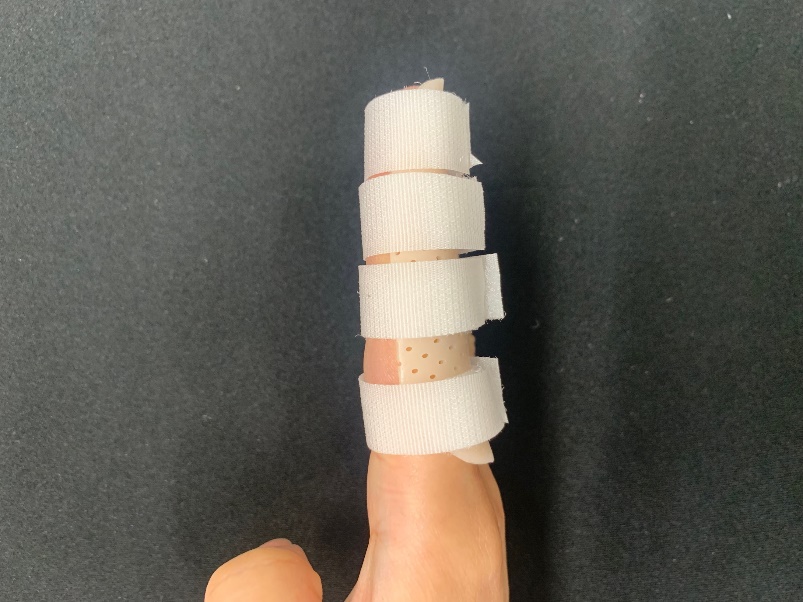 | Fabricate a dorsal thermoplastic orthosis in the maximum comfortable amount of PIPJ extension. Continue to remould splint to increase PIPJ extension | | Day and/or night |
| Capener orthosis | 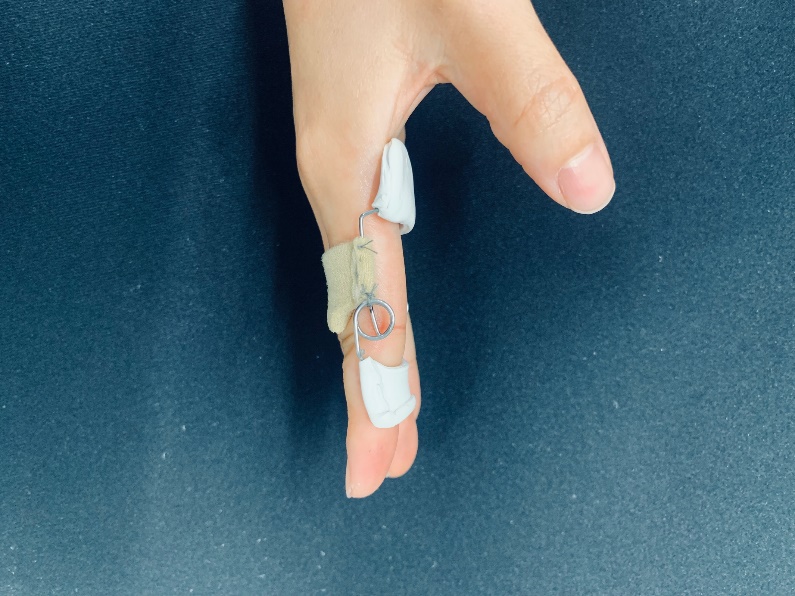 | Fabricate a capener orthosis in the maximum comfortable amount of PIPJ extension | | Day and/or night |
| **Part D:**  **Clinical Scenario Four** | Good passive but reduced active extension | | | |
| Blocked PIPJ extension exercises | 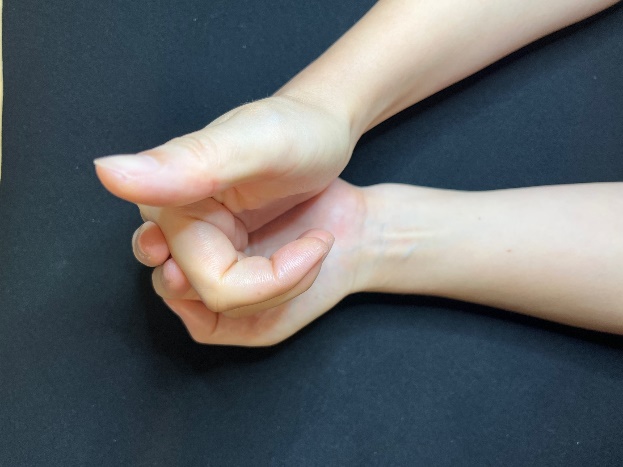 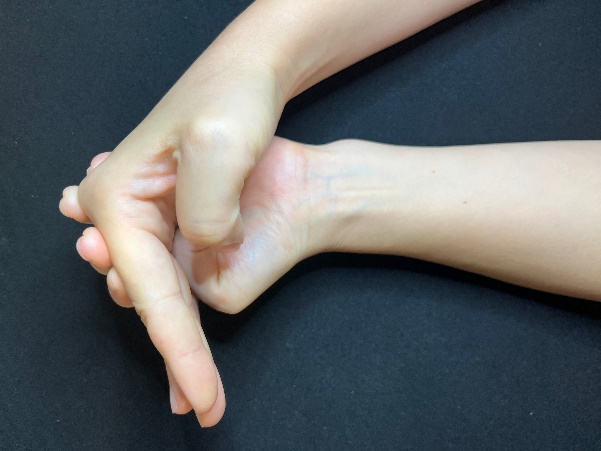 | | | 10 repetitions x 6 sets per  day |
|  | Using the uninjured hand, block the MCPJ’s at maximum flexion and then actively flex and extend the PIPJ and DIPJ while maintaining the flexed position of the MCPJ’s | | |  |
| Resisted extension with  thera-putty | 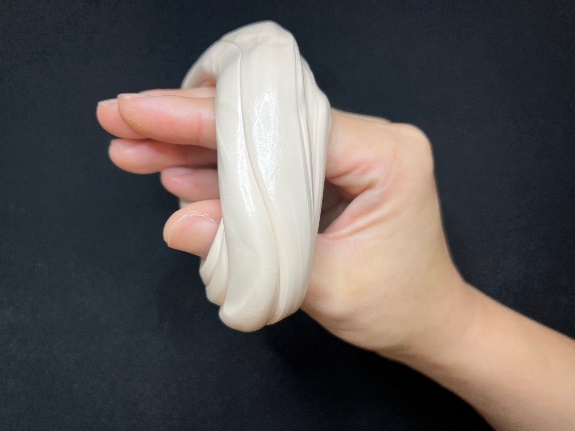 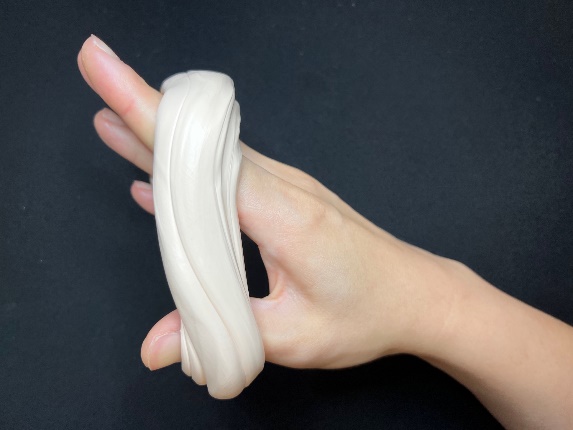 | | | 5-10 mins max  (stop earlier if fatigued) x2/day |
|  | Create a donut shape with the thera-putty and place the digits and thumb inside the thera-putty and then extend the fingers and thumb as far as you can | | |  |
| Gauntlet orthosis | 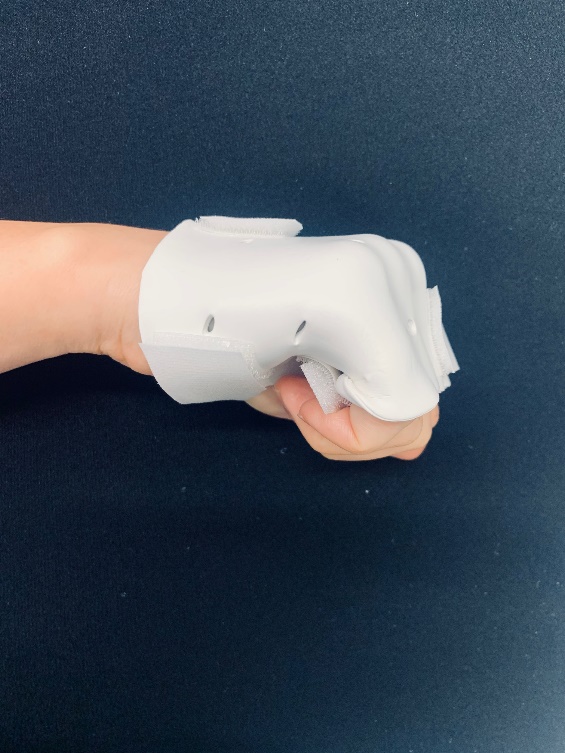 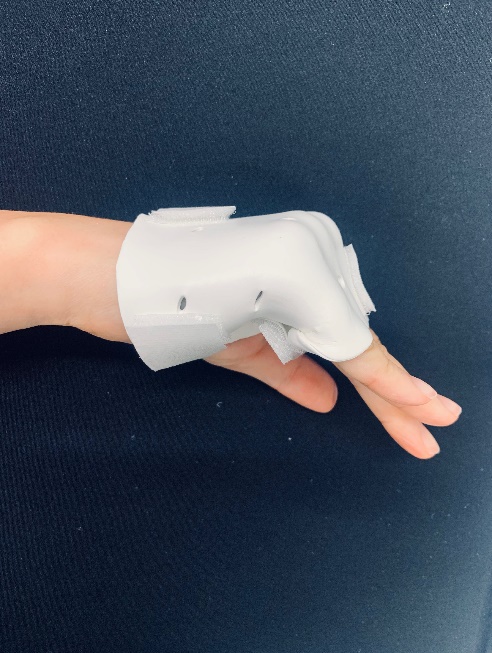 | | | 10 repetitions x 6 sets per day |
|  | Fabricate a thermoplastic orthosis (gauntlet) that positions the MCP’s in maximum flexion and leaves the PIPJ and DIPJ free. Actively flex and extend the PIPJ’s and DIPJ’s within the orthosis. | | |  |
| Relative motion flexion orthosis | 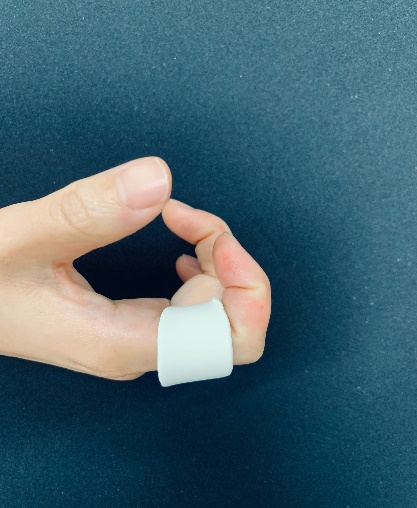 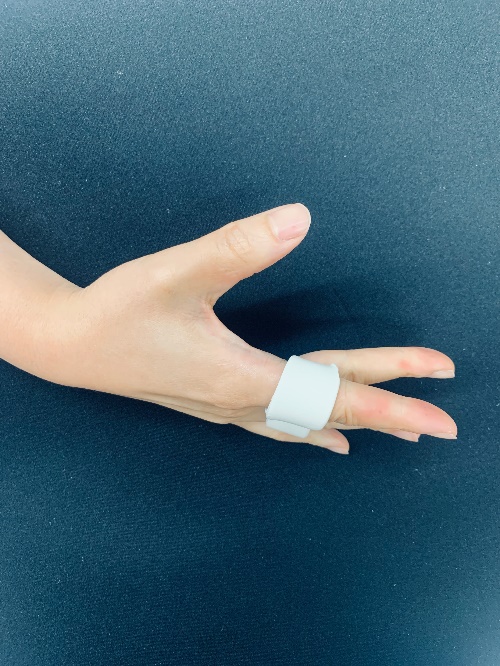 | | | Functional use of hand |
|  | Fabricate a thermoplastic orthosis that positions the injured digit in a position where it is relatively flexed compared to the other digits and use the hand for functional activities while wearing the orthosis | | |  |
